# Effective management of nonconvulsive status epilepticus following cardiac surgery: a case report

**DOI:** 10.1186/s44215-025-00189-3

**Published:** 2025-01-24

**Authors:** Yusuke Yanagino, Taro Yamasumi, Takayuki Miyauchi, Koichi Inoue, Haruhiko Kondoh

**Affiliations:** Department of Cardiovascular Surgery, Japan Organization of Occupational Health and Safety, Osaka Rosai Hospital, Sakai, Osaka 591-8025 Japan

**Keywords:** Nonconvulsive status epilepticus, Status epilepticus, Epilepsy, Seizure, Convulsion

## Abstract

**Background:**

Epileptic seizures following adult cardiovascular surgery occur in 0.9–3% of patients, with the condition in 3–12% of these patients progressing to status epilepticus (SE). SE is a severe condition that significantly impacts prognosis and necessitates early diagnosis and treatment. However, the diagnosis of nonconvulsive status epilepticus (NCSE) is challenging due to its subtle clinical symptoms. Herein, we report a case of NCSE that was diagnosed early by aggressive electroencephalogram (EEG) and treated effectively following cardiac surgery, resulting in discharge without sequelae.

**Case presentation:**

A 44-year-old man with a history of meningitis-induced intellectual disability since childhood underwent aortic valve replacement and grafting of the ascending aorta for a bicuspid aortic valve, severe aortic regurgitation, and ascending aortic dilatation. We observed repeated tonic–clonic seizures on the day of surgery and the following day when the sedation was reduced. On the first postoperative day, an EEG revealed sharp, high-amplitude waves during the tonic–clonic seizure and 2-Hz rhythmic delta activity after motor symptoms disappeared. Based on these findings, the patient was diagnosed with NCSE. Under EEG monitoring, we initially used propofol at 4 mg/kg/h, but owing to a decrease in blood pressure, we achieved deep sedation and burst suppression by combining propofol at 1.5 mg/kg/h with midazolam at 0.18 mg/kg/h. We also administered levetiracetam and fosphenytoin as antiseizure medications. Levetiracetam was administered at 1000 mg/day and fosphenytoin at 20.5 mg/kg, followed by maintenance at 7.2 mg/kg/day. The patient’s consciousness improved upon cessation of sedation on postoperative day 6. Postoperative magnetic resonance imaging revealed no abnormalities. Fosphenytoin was discontinued, and the patient was discharged on postoperative day 32 without any sequelae. The patient continues to take levetiracetam orally at a dose of 1000 mg/day and has been followed up in the outpatient clinic for 4 years without any seizure recurrence.

**Conclusion:**

Postoperative seizures following cardiac surgery may occur with NCSE, even after visible seizures have ceased. This case highlights the importance of thorough EEG monitoring in cases of prolonged disturbance of consciousness, indicating that early diagnosis and treatment of NCSE can improve the prognosis.

## Background

The incidence of epileptic seizures following adult cardiovascular surgery is reported to be between 0.9 and 3% [1–4], with 3–12% cases progressing to status epilepticus (SE) [1, 2]. Generalized convulsive status epilepticus is a severe condition that significantly impacts patient prognosis and presents with prominent motor symptoms, making it relatively straightforward to diagnose [5]. However, nonconvulsive status epilepticus (NCSE), which is also associated with poor prognosis, often presents with subtle clinical symptoms, complicating diagnosis and potentially delaying treatment [5, 6]. The frequency of NCSE in critically ill patients is approximately 10% [7], while after cardiovascular surgery it is reported to be 0.9%. This difference in frequency suggests that some patients may be missed [6]. Furthermore, convulsive seizures reportedly precede NCSE in 42% of cases, and electroencephalogram (EEG) should be considered when seizures stop but consciousness is not regained [6]. In this report, we describe a case of NCSE following a tonic–clonic seizure that was promptly diagnosed through aggressive EEG monitoring, leading to successful treatment and discharge from the hospital without complications.

## Case presentation

**Fig. 1:**
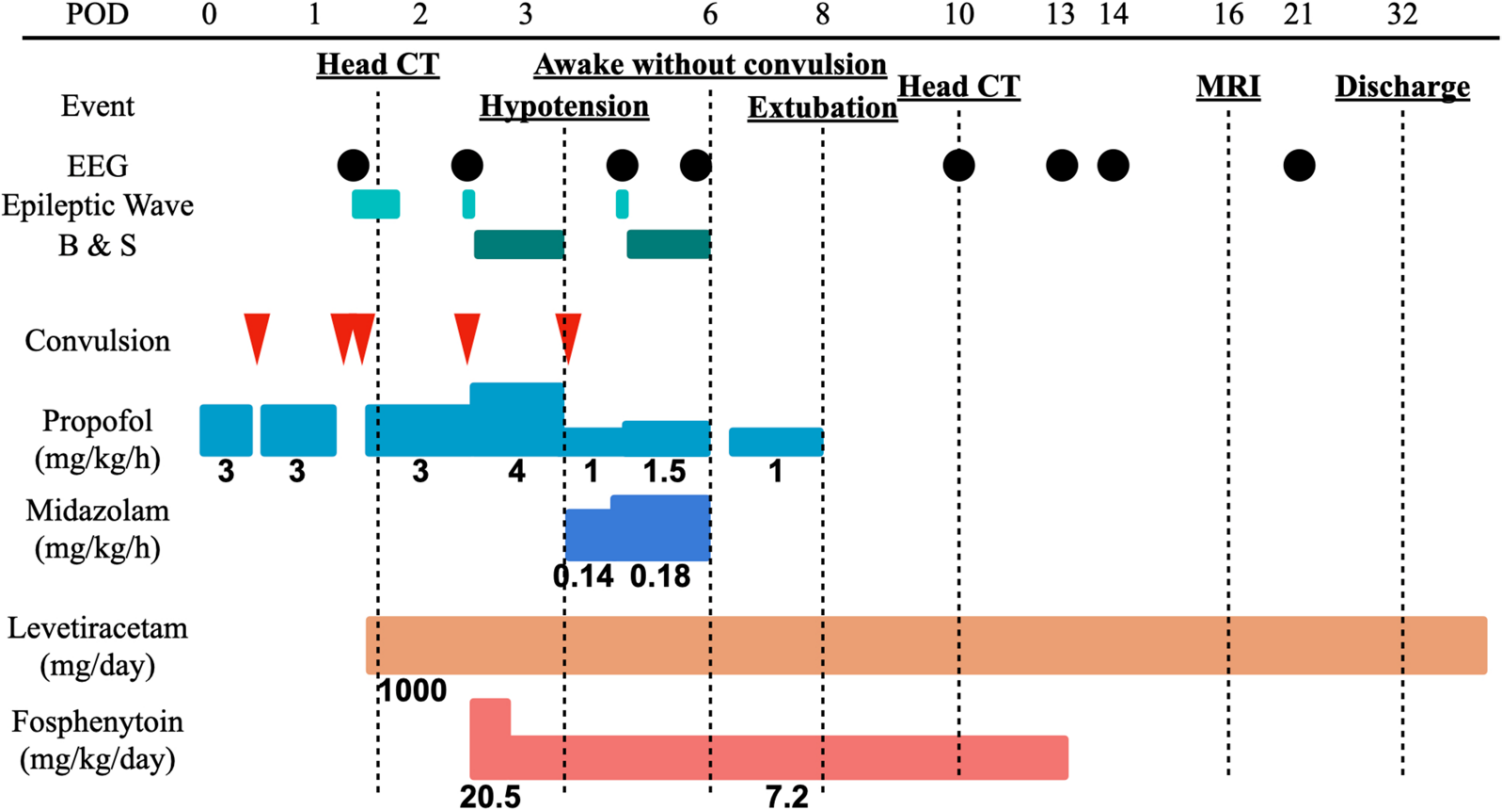
Postoperative progress chart. POD, postoperative day; EEG, electroencephalogram; B and S, burst suppression; CT, computed tomography; MRI, magnetic resonance imaging

**Fig. 2:**
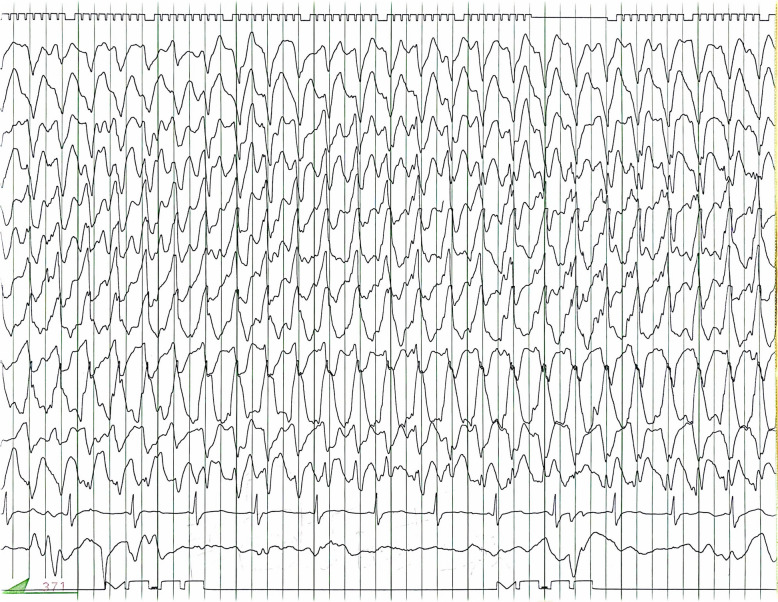
Electroencephalogram during convulsions. Generalized high-amplitude sharp waves are seen

**Fig. 3:**
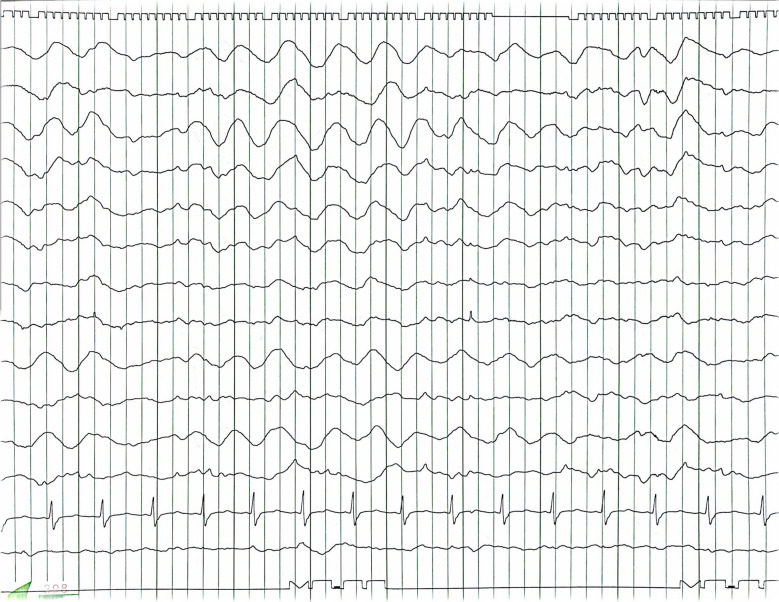
Electroencephalogram after convulsion termination. Generalized 2-Hz slow waves are observed. We consider this to be rhythmic delta activity below 4 Hz, and in combination with the absence of seizures, the patient was diagnosed with nonconvulsive epileptic seizures

**Fig. 4:**
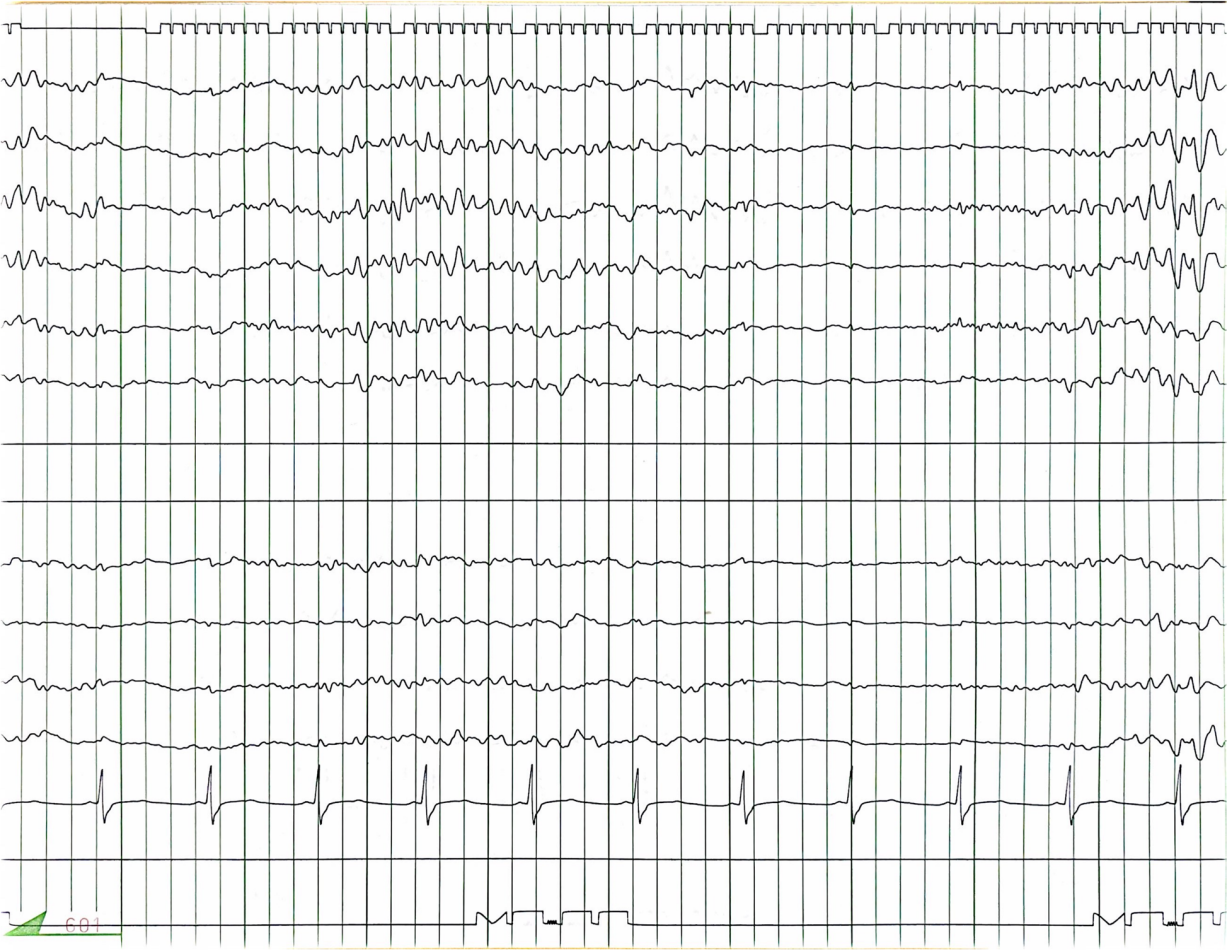
Electroencephalography during deep sedation. Sedation is administered using a combination of propofol and midazolam. Appropriate sedation depth is maintained to achieve burst suppression

A previously healthy 44-year-old man was suspected of having a heart condition during a high school physical examination, but it was not further investigated. Subsequently, a local physician detected a heart murmur, and a transthoracic echocardiography confirmed bicuspid aortic valve (BAV) and severe aortic regurgitation (AR) 2 years before his referral to our hospital. Since he was asymptomatic, he was monitored by his primary care physician. However, 2 years later, his family decided to seek further evaluation at our hospital, prompting his referral. He had a history of meningitis, which resulted in a cognitive deficit (intelligence quotient < 70), with no other significant medical history including seizure. There were no medications in the patient’s prescribed drugs at the time of admission that are known to frequently induce seizures. Transthoracic echocardiography revealed a BAV and severe AR with a left ventricular ejection fraction of 53%, a dilated left ventricular diastolic diameter of 65 mm, and a systolic diameter of 46 mm. Whole-body computed tomography (CT) scans showed an enlarged ascending aorta measuring 45 mm, and no arteriosclerosis was observed in the arteries throughout the body. A head CT scan revealed no abnormalities. Based on these findings, aortic valve replacement (27-mm On-X: CryoLife, Inc., Kennesaw, GA, USA) and ascending aorta grafting (J-graft 1 branch 28 mm: Japan Lifeline Co., Tokyo, Japan) were performed through a median sternotomy using deep hypothermic circulatory arrest (bladder temperature 18.1 °C). Circulatory arrest lasted 24 min, cardiac arrest lasted 159 min, and cardiopulmonary bypass lasted 228 min. The postoperative progress chart is shown in Fig. 1. Tonic–clonic seizures occurred on the day of surgery when sedation was reduced and ceased with further sedation using propofol. Convulsive seizures recurred the next day with reduced sedation, prompting EEG monitoring. Upon the discontinuation of propofol, generalized epileptiform activity started from the right temporal lobe, accompanied by tonic–clonic seizures (Fig. 2). The motor symptoms spontaneously ceased within 2–3 min, but consciousness did not improve, and high-amplitude slow waves resembling rhythmic delta activity persisted (Fig. 3), leading to the diagnosis of NCSE. Head CT showed no signs of bleeding or infarction. Propofol was resumed, and levetiracetam (1000 mg/day) was initiated. On postoperative day 2, seizures were observed when sedation was reduced. The patient was given a loading dose of fosphenytoin 1500 mg (20.5 mg/kg/day), and maintenance of 525 mg/day (7.2 mg/kg/day) was administered, propofol was increased to 4 mg/kg/h under EEG observation, but due to a drop in blood pressure, midazolam was added. Propofol was reduced while maintaining burst suppression on EEG (Fig. 4). On the third postoperative day, generalized high-amplitude sharp waves were observed on EEG without motor symptoms, and the dose of midazolam was increased to maintain burst suppression. On the 6th day, the patient’s consciousness improved without any neurological deficits as sedation was reduced. The patient was extubated on the 7th day postoperatively, fosphenytoin was discontinued on the 13th day, and he was discharged from the intensive care unit. Postoperative magnetic resonance imaging (MRI) showed no organic disease, and levetiracetam at 1000 mg/day was continued. Thereafter, he experienced no major complications and was discharged on postoperative day 32 without sequelae. He discontinued all anticonvulsants 3 years after his discharge and has remained seizure-free for 4 years postoperatively.

## Discussion and conclusions

This study reports an early diagnosis of NCSE, where the patient did not regain consciousness following the resolution of a convulsive seizure, prompting us to suspect NCSE and conduct an EEG. Typically, when motor symptoms appear to have ended, epileptic activity is assumed to have ceased, leading to a lack of EEG testing and missed diagnoses. However, by performing an early EEG in this case, we were able to promptly diagnose and treat the potentially fatal NCSE. Consequently, the patient received appropriate treatment and was discharged without any complications. In cases of nonconvulsive seizures, an EEG is essential for diagnosis but can be time-consuming. However, any delays in conducting an EEG may result in unfavorable outcomes so we have to perform it as early as possible even in the day of surgery to evaluate the effectiveness of drug treatment and to confirm the resolution of the status epilepticus [5, 6]. NCSE is diagnosed using the Saltzburg criteria, necessitating an EEG for confirmation [8–10]. Continuous EEG monitoring immediately postoperatively could benefit patients by supporting early diagnosis, although the low incidence of NCSE may challenge the cost-effectiveness of this approach, making continuous monitoring difficult to justify [3]. In this case, the diagnosis was made without continuous EEG monitoring, as seizures were observed twice within 24 h postoperatively, which prompted EEG testing. This case illustrates that recognizing seizures facilitates diagnosis, although only 42% of patients with NCSE exhibit preceding seizures [6]. Therefore, aggressive EEG testing should be conducted in postoperative cardiac surgery patients with persistent consciousness disorders regardless of the presence or absence of seizures.

Several risk factors for postoperative seizures have been reported after cardiac surgery. Major preoperative risk factors include previous cardiopulmonary arrest, history of neurological disorders, and renal impairment. Surgical factors include aortic surgery and open chamber procedures, whereas intraoperative factors include deep hypothermic circulatory arrest, prolonged cardiopulmonary bypass time (> 150 min), and long cardiac arrest times. Tranexamic acid administration has also been reported as a risk factor [1–4, 11]. However, these risk factors are common in many cardiac surgeries, making the prediction of postoperative seizures challenging. In this case, the patient had multiple risk factors for seizures, including a history of meningitis-related cognitive delay since childhood, aortic surgery under deep hypothermic circulatory arrest, open chamber surgery involving aortic valve replacement, prolonged cardiac arrest (159 min), and a long cardiopulmonary bypass time (228 min). However, the risk factors for postoperative NCSE are poorly defined, and reports are rare. Therefore, further accumulation and analysis of these cases are necessary to clarify their causes and risk factors.

Treatment of SE involves seizure suppression and adequate sedation [12]. If SE continues for more than 5 min, the guidelines of the American Epilepsy Society suggest the management of the respiratory and circulatory systems, types and doses of anticonvulsants and sedatives, and timing of blood tests, CT, MRI, EEG, and cerebrospinal fluid examinations [12]. Moreover, reports suggest that maintaining deep sedation and achieving a flat EEG or a burst suppression pattern on the EEG lead to better outcomes in SE [13]. Despite the suppression of seizures, EEG was used to confirm the therapeutic effects, facilitating the diagnosis of NCSE. Additionally, adjusting the depth of sedation to achieve burst suppression under EEG monitoring over 3 days allowed for a gradual recovery of consciousness, enabling discharge without neurological abnormalities. Therefore, even in the absence of obvious seizures, we can easily detect the epileptiform discharge and provide appropriate treatment, by using the EEG test for patients with NCSE. Head imaging is recommended for diagnosis, but there is no clear answer as to which imaging test should be prioritized. However, recent advancements in CT imaging, combined with contrast-enhanced CT, make it possible to diagnose urgent conditions such as cerebral infarction and cerebral hemorrhage that require immediate intervention. MRI imaging immediately after cardiovascular surgery can be challenging, and, as in this case, it may be sufficient to perform imaging once the patient’s overall condition has stabilized.

This report represents only a single case and cannot be generalized to all cases. However, reports of NCSE following cardiac surgery are rare [6]. We believe that this paper, which provides a detailed account of the course of the condition, offers valuable insights to cardiac surgeons and intensivists.

In conclusion, we report a case of nonconvulsive SE following cardiac surgery. Even when convulsive seizures cease, NCSE may occur; thus, aggressive EEG testing is necessary in cases of delayed recovery of consciousness. If NCSE is diagnosed, adjusting and maintaining the depth of sedation under continuous EEG monitoring to achieve burst suppression are an effective treatment.

## Data Availability

Not applicable.

## Abbreviations

SE: Status epilepticus
NCSE: Nonconvulsive status epilepticus
EEG: Electroencephalogram
CT: Computed tomography
MRI: Magnetic resonance imaging
ICU: Intensive care unit
